# Expression of autophagy-related protein beclin-1 in malignant canine mammary tumors

**DOI:** 10.1186/1746-6148-9-75

**Published:** 2013-04-11

**Authors:** Jing-Lan Liu, Kai-Chian Chang, Chun-Chih Lo, Pei-Yi Chu, Chen-Hsuan Liu

**Affiliations:** 1 Department of Pathology, St. Martin De Porres Hospital, No. 565, Section 2, Daya Road, Chiayi, 60069, Taiwan; 2 School of Medicine, Fu-Jen Catholic University, No. 510, Zhongzheng Road, Xinzhuang Dist., New Taipei City, 24205, Taiwan; 3 Graduate Institute of Molecular and Comparative Pathobiology, School of Veterinary Medicine, National Taiwan University, No. 1, Section 4, Roosevelt Road, Taipei, 10617, Taiwan

**Keywords:** Autophagy, Beclin-1, Canine mammary tumor, Immunohistochemistry

## Abstract

**Background:**

Autophagy is a self-catabolic mechanism that degrades unnecessary cellular components through lysosomal enzymes. Beclin-1, an autophagy-related protein, establishes the first connection between autophagy and tumorigenesis. The purpose of this study is to assess the Beclin-1 expression pattern and to determine its prognostic significance in patients with malignant canine mammary tumor (CMT).

**Results:**

We examined Beclin-1 expression in 70 cases of malignant CMTs by immunohistochemistry. Cytoplasmic Beclin-1 expression was significantly weaker in cancer cells than in nearby normal mammary glands (*p* < 0.001). Low cytoplasmic expression (57.14%) was associated with older age, lower degree of tubular formation, increased mitotic activity, higher histologic grade, and extensive necrosis. Low nuclear expression (40%) was connected with older age, lower degree of tubular formation, extensive necrosis, and negative for Her2/neu overexpression. Univariate survival analysis showed that Beclin-1 cytoplasmic expression was a poor prognostic factor for overall survival rate (*p* < 0.001). Multivariate survival analysis demonstrated that Beclin-1 cytoplasmic expression is an independent prognostic factor (*p* = 0.016).

**Conclusions:**

Loss of Beclin-1 is associated with aggressive clinicopathologic features and poor overall survival. The results suggest that Beclin-1 plays an important role in tumor progression of malignant CMTs.

## Background

Canine mammary tumors (CMTs) are the most common neoplasms in intact female dogs. Approximately half of CMTs are malignant. Histologically, the majority of malignant CMTs are carcinomas, whereas approximately 10% are sarcomas. The spontaneously occurring malignant CMTs share many clinicopathologic and molecular characteristics with human breast cancers. The comparative analysis of human and dog genomes demonstrates the similarity of orthologous genes between the 2 species [1,2]. Therefore, malignant CMTs can be used as a suitable animal model for oncogenesis research and treatment protocols.

Programmed cell death is a genetically mediated process via internal or external signal pathways. Two types of programmed cell death, apoptosis and autophagy, have been subjects of increasing attention to scientists. Apoptosis involves the activation of catabolic enzymes in the signaling transduction pathway that leads to self-destruction. The term “autophagy” was first introduced in 1963 by de Duve, the discoverer of lysosomes [3]. Autophagy is a self-catabolic process that involves the degradation of intracellular structures and organelles by lysosomal enzymes [4]. Autophagy is essential for development, homeostasis, and survival, especially for stress adaption in an energy-deficient environment. It is also closely related to many pathologic processes, such as infections, metabolic disorders, neurodegeneration, and tumorigenesis [5].

Autophagy is regulated by a group of evolutionarily conserved genes, which were first discovered in yeast [6]. To date, more than 30 autophagy-related genes have been identified. The *BECN1* gene is the mammalian orthologue of the yeast *apg6/vps30*, and was the first gene to establish a connection between autophagy and tumorigenesis [7]. Two research groups have shown that *BECN1* heterozygous-deficient mice have a higher frequency of spontaneous tumors, whereas homozygous-deficient mice died early in embryogenesis because of defects in proamniotic canal closure [8,9]. They concluded that *BECN1* is a haplo-insufficient tumor suppressor gene. The Beclin-1 protein, which is encoded by the *BECN1* gene, is involved in the signaling pathway of autophagy and is required for the nucleation of the phagophore and maturation of the autolysosome. Beclin-1 expression can indicate autophagic activity in cells. Beclin-1 expression and its association with clinicopathologic features have not been described in canine cancer. The aims of the study were to compare Beclin-1 expression patterns in normal mammary glands and malignant CMTs, to investigate the clinicopathologic significance of Beclin-1 expression, and to evaluate its impact on clinical outcomes.

## Results

### Patient characteristics

This study comprised 70 cases of malignant CMTs, including 54 simple carcinomas, 11 complex carcinomas, and 5 sarcomas. The mean age of 69 dogs at the time of surgery was 11.3 ± 2.7 years (ranging from 4 to 18 years). The age of the remaining dog was unknown. In total, 16 of 70 (22.9%) dogs received ovario-hysterectomy prior to the surgical removal of tumors. The mean maximum tumor size was 4.3 ± 3.1 cm (ranging from 0.4 to 15.0 cm). The other clinicopathologic features, including tumor location, tubular formation, nuclear pleomorphism, mitotic count, histologic grade, lymphovascular invasion, necrosis, expressions of estrogen receptor and Her2, were summarized in Table 1.

**Table 1:** Association of Beclin-1 expression pattern and clinicopathologic variables in 70 cases of malignant CMTs

		**Beclin-1 cytoplasmic expression**		***p *value**	**Beclin-1 nuclear expression**		***p *value**
**Variable**	**No. of cases**	**Low**	**High**		**Low**	**High**	
Age^a^							
≦ 11 years	36	15 (38.5%)	21 (70.0%)	0.009*	10 (37.0%)	26 (61.9%)	0.044*
> 11 years	33	24 (61.5%)	9 (30.0%)		17 (63.0%)	16 (38.1%)	
Location of affected gland							
Cranial gland	24	14 (35.0%)	10 (33.3%)	0.953	9 (32.1%)	15 (35.7%)	0.890
Caudal gland	42	24 (60.0%)	18 (60.0%)		17 (60.7%)	25 (59.5%)	
Both	4	2 (5.0%)	2 (6.7%)		2 (7.1%)	2 (4.8%)	
Tumor size							
≦ 3 cm	27	13 (32.5%)	14 (46.7%)	0.228	9 (32.1%)	18 (42.9%)	0.367
> 3 cm	43	27 (67.5%)	16 (53.3%)		19 (67.9%)	24 (57.1%)	
Histologic classification							
Simple carcinoma	54	30 (75.0%)	24 (80.0%)	0.877	23 (82.1%)	31 (73.8%)	0.640
Complex carcinoma	11	7 (17.5%)	4 (13.3%)		3 (10.7%)	8 (19.0%)	
Sarcoma	5	3 (7.5%)	2 (6.7%)		2 (7.1%)	3 (7.1%)	
Tubular formation							
> 10% of the tumor	45	19 (47.5%)	26 (86.7%)	0.001*	14 (50.0%)	31 (73.8%)	0.042*
≦ 10% of the tumor	25	21 (52.5%)	4 (13.3%)		14 (60.0%)	11 (26.2%)	
Nuclear pleomorphism							
Mild to moderate	46	17 (42.5%)	19 (63.3%)	0.084	12 (42.9%)	24 (57.1%)	0.241
Marked	34	23 (57.5%)	11 (36.7%)		16 (57.1%)	18 (42.9%)	
Mitotic count							
≦10/10 HPFs	52	25 (62.5%)	27 (90.0%)	0.009*	18 (64.3%)	34 (81.0%)	0.118
>10/10 HPFs	18	15 (37.5%)	3 (10%)		10 (35.7%)	8 (19.0%)	
Histologic grade							
Grades 1 and 2	56	27 (67.5%)	29 (96.7%)	0.003*	18 (64.3%)	38 (90.5%)	0.007*
Grade 3	14	13 (32.5%)	1 (3.3%)		10 (35.7%)	4 (9.5%)	
Lymphovascular invasion							
Absent	51	26 (65.0%)	25 (83.3%)	0.088	19 (67.9%)	32 (76.2%)	0.442
Present	19	14 (35.0%)	5 (16.7%)		9 (32.1%)	10 (23.8%)	
Necrosis							
Limited/no necrosis	41	17 (42.5%)	24 (80%)	0.002*	12 (42.9%)	29 (69.0%)	0.029*
Extensive necrosis	29	23 (57.5%)	6 (20%)		16 (57.1%)	13 (31.0%)	
Estrogen receptor							
Negative	25	16 (40%)	9 (30%)	0.388	9 (32.1%)	16 (38.1%)	0.611
Positive	45	24 (60%)	21 (70%)		19 (67.9%)	26 (61.9%)	
Her2 overexpression							
Negative	51	29 (72.5%)	22 (73.3%)	0.938	24 (85.7%)	27 (64.3%)	0.048*
Positive	19	11 (27.5%)	8 (26.7%)		4 (14.3%)	15 (35.7%)	

HPF, High power field.

^a^The age of one case is unknown.

*p < 0.05.

### Comparison of Beclin-1 expression in normal mammary glands and cancer cells

**Figure 1:**
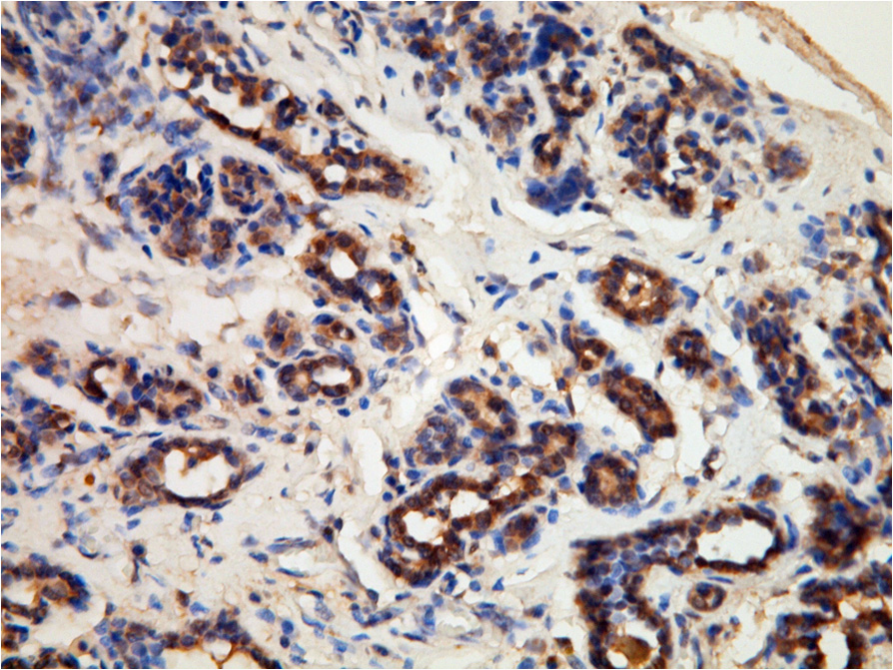
**Beclin-1 expression in normal mammary glands of dogs.** The normal mammary glands show diffuse moderate cytoplasmic expression and scattered nuclear expression.

**Figure 2:**
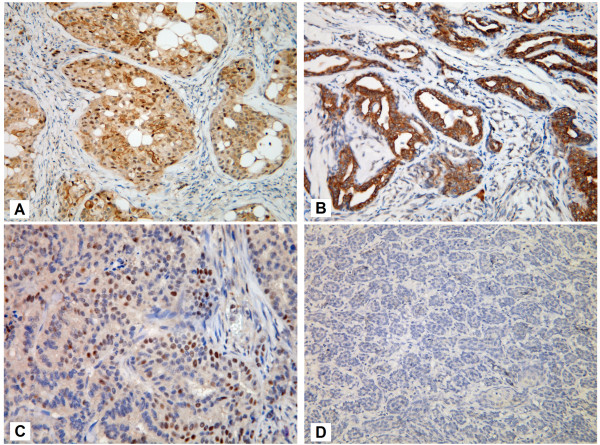
**Beclin-1 expression patterns in malignant CMTs.** (**A**) High cytoplasmic and high nuclear expression pattern. (**B**) High cytoplasmic and low nuclear expression pattern. (**C**) Low cytoplasmic and high nuclear expression pattern. (**D**) Low cytoplasmic and low nuclear expression pattern.

**Figure 3:**
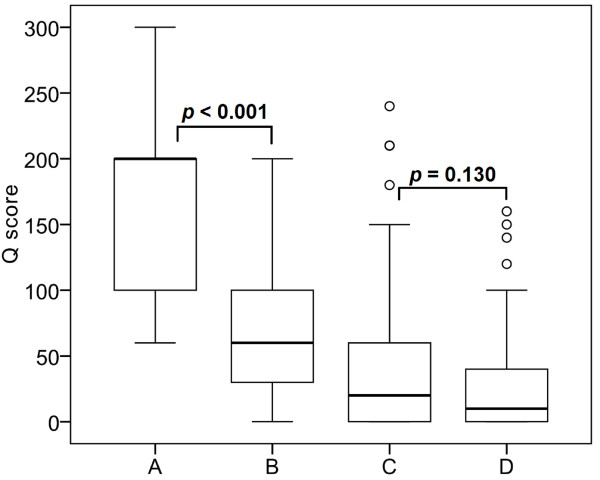
**Comparison of Q scores of Beclin-1 in normal mammary glands and malignant CMTs. A**. Cytoplasmic expression in normal mammary glands. **B**. Cytoplasmic expression in malignant CMTs. **C**. Nuclear expression in normal mammary glands. **D**. Nuclear expression in malignant CMTs. The middle lines of boxes show the median value, whereas top and bottom lines of boxes represent interquartile range. The ends of whiskers represent 10th and 90th percentiles. The outliers are indicated by open circles.

The normal mammary glands near the cancer cells showed weak or moderate cytoplasmic reactivity and variable nuclear expression of Beclin-1 (Figure 1). The cancer cells displayed negative, weak, or moderate cytoplasmic staining, and ranged from non-reactivity to strong positivity of nuclear expression (Figure 2). The cytoplasmic Q score of normal mammary glands was significantly higher than that of cancer cells (*p* < 0.001). The difference of nuclear Q score between normal glands and cancer cells was not statistically significant (*p* = 0.130) (Figure 3).

### Association of Beclin-1 expression in cancer cells and clinicopathologic characteristics

The associations between Beclin-1 expression patterns and clinicopathologic variables are shown in Table 1. The median value of the Q score of cytoplasmic Beclin-1 expression in malignant CMTs was 60. Using the median value as a cutoff point, 40 cases (57.14%) were classified as low cytoplasmic expression, whereas 30 cases (42.86%) were classified as high cytoplasmic expression. Low cytoplasmic expression (Q score ≦60) of Beclin-1 was associated with older age, lower degree of tubular formation, increased mitotic activity, higher histologic grade, and extensive necrosis. The median value of the nuclear Q score in malignant CMTs was 10. In total, 28 cases (40%) were sub-grouped into low nuclear expression, and 42 cases (60%) were sub-grouped into high nuclear expression. Low nuclear expression (Q score ≦10) of Beclin-1 was linked to older age, lower degree of tubular formation, extensive necrosis, and negative for Her2/neu overexpression. Beclin-1 cytoplasmic expression was linked significantly with nuclear expression (*p* = 0.003) (Table 2).

**Table 2:** Association of nuclear expression and cytoplasmic expression of Beclin-1 in 70 cases of malignant CMTs

		**Nuclear expression**		
		**Low**	**High**	***p *value**
Cytoplasmic expression	Low	22 (31.43%)	18 (25.71%)	0.003*
	High	6 (8.57%)	24 (34.29%)	

* *p* < 0.05.

### Survival analysis

**Figure 4:**
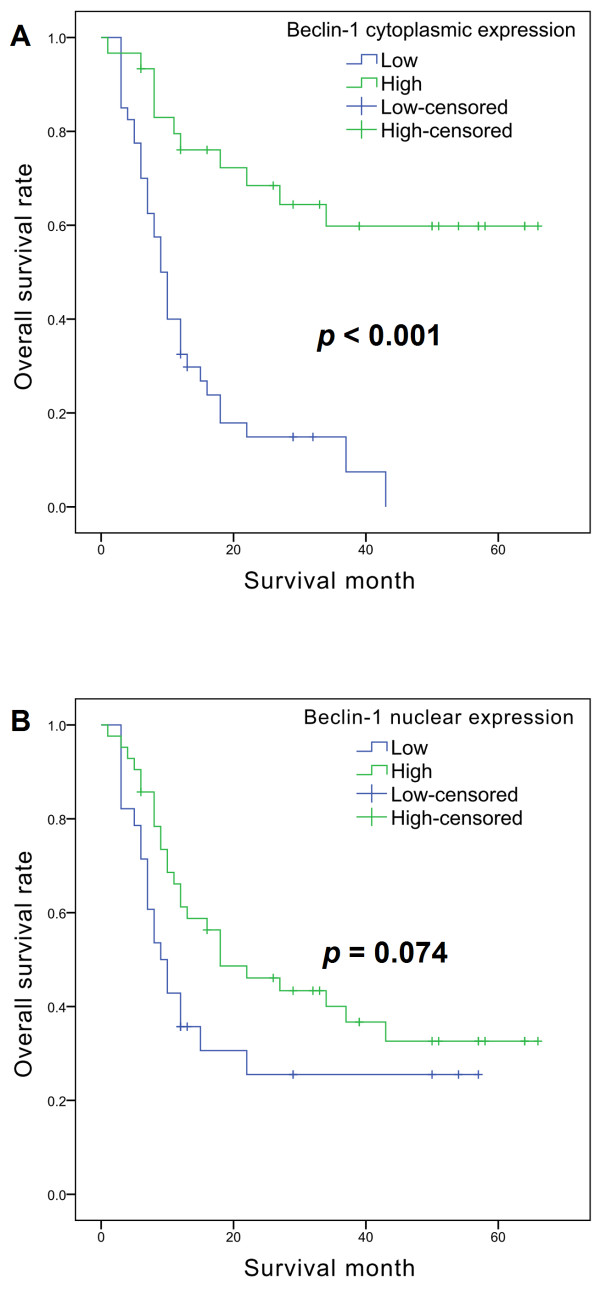
**Kaplan-Meier curves of overall survival rate in 70 cases of malignant CMTs.** (**A**) Curves for patients with low and high cytoplasmic Beclin-1 expressions. (**B**) Curves for patients with low and high nuclear Beclin-1 expressions.

The mean follow-up time was 21 ± 18.72 months. Univariate survival analysis using the Kaplan-Meir method revealed that age, tumor size, tubular formation, nuclear pleomorphism, mitotic count, histologic grade, lymphovascular invasion, necrosis, and Beclin-1 cytoplasmic expression were significant prognostic factors for overall survival (Table 3). Figure 4 shows the Kaplan-Meier curves of cumulative overall survival probability in relation to the Beclin-1 expression of cancer cells. Patients with low cytoplasmic expression showed poorer overall survival rate (*p* < 0.001). The difference of overall survival rate between high and low nuclear expressions was not statistically significant (*p* = 0.074). Multivariate survival analysis using the Cox proportional hazard regression method revealed that tumor size, tubular formation, and Beclin-1 cytoplasmic expression were independent prognostic factors for malignant CMTs (Table 4).

**Table 3:** Univariate analysis of clinicopathologic variables for ovarall survival rate

**Variables**	***p *value**
Age (> 11 years vs. ≦ 11 years)	0.001*
Tumor size (> 3 cm vs. ≦ 3 cm)	0.042*
Tubular formation (> 10% vs. ≦ 10%)	<0.001*
Nuclear pleomorphism (high vs. low)	0.044*
Mitotic count (> 11 vs. ≦ 10/10 HPFs)	0.005*
Histologic grade (3 vs. 2 and 1)	<0.001*
Lymphovascular invasion (yes vs. no)	<0.001*
Necrosis (extensive vs. limited/no)	0.004*
Estrogen receptor (negative vs. positive)	0.180
Her2/neu overexpression (positive vs. negative)	0.348
Beclin-1 cytoplasmic expression (low vs. high)	<0.001*
Beclin-1 nuclear expression (low vs. high)	0.074

* *p* < 0.05.

**Table 4:** Multivariate Cox proportional hazard analysis of clinicopathologic variables for ovarall survival rate

	**Multivariate analysis**		
	**Hazard ratio**	**95% confidence interval**	***p *value**
Age (> 11 years vs. ≦ 11 years)	1.460	0.683 - 3.124	0.329
Tumor size (> 3 cm vs. ≦ 3 cm)	3.038	1.298 - 7.108	0.010*
Tubular formation (> 10% vs. ≦ 10%)	0.364	0.167 - 0.794	0.011*
Nuclear pleomorphism (high vs. low)	1.001	0.445 - 2.253	0.997
Mitotic count (> 11 vs. ≦ 10/10 HPFs)	0.881	0.166 - 4.676	0.882
Histologic grade (3 vs. 2 and 1)	0.627	0.088 - 4.486	0.642
Lymphovascular invasion (yes vs. no)	2.286	0.887 - 5.887	0.087
Necrosis (extensive vs. limited/no)	0.977	0.473 - 2.019	0.951
Estrogen receptor (negative vs. positive)	0.499	0.232 - 1.076	0.076
Her2/neu overexpression (positive vs. negative)	2.433	0.950 - 6.234	0.064
Beclin-1 cytoplasmic expression (low vs. high)	2.752	1.211 - 6.256	0.016*
Beclin-1 nuclear expression (low vs. high)	1.471	0.667 - 3.243	0.339

* *p* < 0.05.

## Discussion

Autophagy, an essential catabolic mechanism, is also involved in tumor initiation and progression. Recent studies have revealed that the expression of Beclin-1 is decreased in various human cancer types, such as breast [10], cervical [11], esophageal [12], lung cancers [13,14], hepatocellular carcinoma [15], and cutaneous melanoma [16]. However, Beclin-1 expression was reported to be increased in human colon, gastric, and pancreatic cancers, in contrast to their normal counterparts [17,18]. The mechanism of aberrance of Beclin-1 expression in different types of cancers is largely unknown. These variable results imply that autophagic activity is specific in different organs and histologic types. They also indicate that autophagy may either induce or inhibit tumor cell survival. In this study, we compared the Beclin-1 expression in malignant CMTs and surrounding normal mammary glands. Cytoplasmic expression of cancer cells was significantly lower than that of normal mammary glands. Decreased expression of Beclin-1 was associated with some aggressive histologic features. These findings were similar to those of human breast cancer [7,10]. Malignant CMT has similar epidemiologic, histologic, clinical, and prognostic features to human breast cancer. Our results imply that the autophagic activities in canine and human mammary glands may also be coincidental. Further comparative studies of autophagy may be beneficial to both human beings and dogs.

The subcellular localization of Beclin-1 was mainly reported at the cytoplasm and/or membrane, and the nuclear expression pattern was also documented [19,20]. The leucine-rich nuclear export signal of Beclin-1 is essential for autophagic growth control and tumor suppression [21]. Our study disclosed that nuclear expression is associated with cytoplasmic expression. Lower nuclear expression is also related to unfavorable clinicopathologic features.

The relationship between the expression pattern of Beclin-1 and the prognosis was controversial in studies of human cancer. Loss of Beclin-1 was linked to poorer survival rate in stage III colon cancer [19], esophageal squamous cell carcinoma [12], hepatocellular carcinoma [15], intrahepatic cholangiocarcinoma [22], pancreatic ductal adenocarcinoma [18], chondrosarcoma [23], and several types of lymphoma [24-26]. High Beclin-1 expression was connected to poor prognosis in endometrial adenocarcinoma [27] and nasopharyngeal carcinoma in humans [28]. Koukourakis et al. found that extensive overexpression and underexpression of Beclin-1 was associated with poor overall survival in human patients with colon cancer [20]. They noted that the nuclear expression of Beclin-1 was not related to the prognosis. These results indicate that Beclin-1 may either induce or inhibit tumor cell survival. Our results support the hypothesis that Beclin-1 functions as a tumor suppressor protein in malignant CMTs. The mechanisms by which autophagy suppresses and promotes carcinogenesis are not yet completely understood.

Both autophagy promoters and autophagy inhibitors are clinically effective in cancer treatment [29-32]. The autophagic tumor stroma model of cancer proposed by Martinez-Outschoorn et al. attempted to resolve the paradox [33]. In this model, cancer cells use oxidative stress to induce autophagy in the tumor environment, whereas the autophagic tumor stromal cells produce recycled nutrients to promote the growth of cancer cells [34]. Sanchez et al. discovered that the mesenchymal stem cell-derived stromal cells in human breast cancer showed upregulation of Beclin-1 and other autophagic markers [35]. However, this model may not explain the upregulation of autophagy-related proteins in some human cancer cells. Our study and other previous researches did not find a specific immunohistochemical staining pattern of Beclin-1 in cancer-associated stromal cells. Moreover, the Beclin-1 independent autophagic process may also be considered. More proteomic-based studies should be performed to clarify the functions of autophagy-related proteins in cancer and cancer-associated stromal cells.

## Conclusions

We analyzed the Beclin-1 expression pattern in normal mammary glands and malignant CMTs. We found that the loss of Beclin-1 expression is associated with aggressive clinicohistologic features and poor overall survival. Our results suggest that Beclin-1 plays a significant role in tumor progression and can be a potential therapeutic target for malignant CMTs in the future.

## Methods

### Patients and tissue samples

Formalin-fixed, paraffin-embedded tissue samples from 70 female dogs with primary malignant CMTs were analyzed in this study. The 70 dogs included 21 mongrels, 19 Maltese, 7 Shih-Tzus, 6 poodles, 4 Cocker spaniels, 4 Schnauzers, 4 Yorkshire terriers, 2 Labrador retrievers, 1 French spaniel, 1 Pomeranian, and 1 spitz. All of these specimens were surgically resected at National Taiwan University Veterinary Hospital from 2005 to 2011. Patients who received chemotherapy before or after surgery were excluded from this study. All cases were pathologically diagnosed with primary malignant CMTs at the School of Veterinary Medicine, National Taiwan University. Information such as age, breed, status of ovario-hysterectomy, and tumor size of patients was obtained from medical records. Follow-up data were obtained from medical records and by telephone contact with the dog owners. Overall survival was defined as the time between surgery and death.

### Pathologic examination

Routine hematoxylin and eosin (HE) staining was performed for histologic assessment. The histologic type was assessed according to the diagnostic criteria of the World Health Organization Histological Classification of Mammary Tumors of the Dog and Cat [36]. The tumors were graded based on Nottingham Modification of the Bloom-Richardson system on HE-stained sections [37,38]. The grading system combined 3 histopathologic features: tubular formation, nuclear pleomorphism, and mitotic counts. Each feature was scored 1 to 3 points. The histologic grade was according to the final scores as follows: 3, 4, or 5 points as grade I (well differentiated); 6 or 7 points as grade II (moderately differentiated); and 8 or 9 points as grade III (poorly differentiated). Necrotic areas of more than 10% of the tissue section were regarded as “extensive,” whereas the remaining cases were recorded as “limited/no” necrosis. The presence of cancer cells within vascular channels of the primary tumor was detected in HE–stained sections.

### Immunohistochemical staining

Tissue blocks containing representative cancer areas with surrounding normal breast tissue were selected from every case. Immunohistochemical staining was performed on 4-μm-thick tissue sections using the Leica Bond-Max autostainer (Leica microsystem) according to the manufacturer’s instructions with minor modifications. Sections were deparaffinized by the Bond Dewax Solution (Leica Microsystems). Heat-induced antigen retrieval was achieved using the Bond Epitope Retrieval Solution 2 (Leica Microsystems) for 20 min at 100°C. Endogenous peroxidase activity was blocked by incubation of tissue with the Novocastra Peroxidase Block for 5 min. The slides were then incubated for 30 min at room temperature with a primary rabbit polyclonal antibody against Beclin-1 (1: 400, Abcam, UK), estrogen receptor (1:50, clone 6 F11, Novocastra, UK), and Her2 (1:50, clone CB11, Novocastra, UK). Diaminobenzidine-tetrahydrochloride (DAB) was used as the substrate to detect antigen-antibody binding. Sections were counterstained with hematoxylin.

### Immunohistochemical evaluation

For Beclin-1, the intensity, percentage, and sub-localization of immunohistochemical stains in cancer cells and cancer-adjacent normal mammary glands of each case were recorded. The normal canine mammary ductal epithelium from dogs without mammary neoplasia was used as positive control, and staining with omission of primary antibody was performed as negative control. The intensity and percentage of positively stained cells were counted at 10 high-powered fields (400 ×). The intensity of staining was recorded as 0, 1, 2, and 3 standing for negative, weak, moderate and strong staining, respectively. The percentage of positive cells was scored from 0% to 100%. The results of cytoplasmic and nuclear Beclin-1 expressions were scored by quick score (Q), which is obtained by multiplying the percentage of positive cells (P) by the intensity (I) (Q = P × I; maximum = 300) [39]. The median value of the Q score in cancer cells was used as a cutoff point, and the cases were sub-grouped into “low expression” and “high expression”.

The Her2 expression was scored according to the American Society of Clinical Oncology/College of American Pathologists guidelines (0 = no staining or membrane staining in fewer than 10% of tumor cells; 1 + = faint, barely perceptible membrane staining in more than 10% of tumor cells; 2 + = weak to moderate complete membrane staining observed in more than 10% of tumor cells or strong complete membrane staining in less than 30% of tumor cells; 3 + = strong and complete membrane staining in more than 30% tumor cells) [40]. In this study, Her2 positive was defined as a score of 3+, whereas the rest were regarded as negative. For estrogen receptor, nuclear staining more than 10% of cancer cells were classified as positive, while the others were classified as negative.

Immunohistochemical staining was evaluated by two pathologists independently without knowledge of clinical outcomes of the patients. Conflicting results were resolved at multi-headed microscope.

### Statistical analysis

The Wilcoxon signed-rank test was used to analyze Beclin-1 expression in paired normal mammary glands and cancer cells. The chi-square test was used to evaluate the association of Beclin-1 expression with clinicopathologic features of malignant CMTs. Curves for overall survival were drawn using the Kaplan-Meier method, and the differences of survival rate were compared using the log-rank test for univariate survival analysis. The Cox proportion hazard regression model was used for multivariate survival analysis of prognostic factors. A *p* value of less than 0.05 was considered statistically significant. The statistical analysis was performed by SPSS 19.0 software in Windows.

## Abbreviations

CMT: Canine mammary tumor; HE: Hematoxylin and eosin.

## Competing interests

The authors declare that no competing interests exist.

## Authors’ contributions

J-LL analyzed the data, performed statistical analyses, and drafted the manuscript. K-CC and C-CL performed immunohistochemical assay and participated in the interpretation of the data. P-YC and C-HL designed and directed the studies, and critically revised the manuscript. All authors have read and approved of the final version of the manuscript.
